# Safety and effectiveness of minimally invasive sacroiliac joint fusion in women with persistent post-partum posterior pelvic girdle pain: 12-month outcomes from a prospective, multi-center trial

**DOI:** 10.1186/s40064-015-1359-y

**Published:** 2015-10-05

**Authors:** Robyn Capobianco, Daniel Cher, for the SIFI Study Group

**Affiliations:** SI-BONE, Inc., 3055 Olin Ave Suite 2200, San Jose, CA 95128 USA

**Keywords:** Sacroiliac joint fusion, Postpartum pelvic girdle pain, Sacroiliac joint dysfunction, Sacroiliac joint disruption, Degenerative sacroiliitis

## Abstract

Postpartum posterior pelvic girdle pain (PPGP) affects nearly 20 % of women who experience back pain in the peripartum period. The sacroiliac joint is a source of this pain in 75 % of women with persistent PPGP. A subset of women will fail to obtain acceptable pain relief from the current array of non-surgical treatment options. The purpose of this study is to assess the safety and effectiveness of minimally invasive sacroiliac (SI) joint fusion in women with chronic SI joint dysfunction whose pain began in the peri-partum period whose symptoms were recalcitrant to non-surgical management. A sub-group analysis of subjects with sacroiliac joint disruption and/or degenerative sacroiliitis enrolled in a prospective, multi-center trial of SI joint fusion was performed. Subjects with PPGP were identified and compared with women without PPGP and with men. Of 172 enrolled subjects, 52 were male, 100 were females without PPGP and 20 females had PPGP. PPGP subjects were significantly younger (43.3 years, vs. 52.8 for females without PPGP and 50.5 for men, p = 0.002). There were no differences in any other demographic or baseline clinical measure. Women with PPGP experienced a significant improvement in pain (−51 mm on VAS), function (−20.6 pts on ODI) and quality of life (SF-36 PCS +10.4, MCS +7.2, EQ-5D +0.31) at 12 months after surgery. These improvements were characteristic of the overall study results; no difference was detected between sub-groups. The sacroiliac joint can be a source of pain in women with persistent PPGP and should be investigated as a pain generator. In this study, women with carefully diagnosed chronic SI joint pain from PPGP recalcitrant to conservative therapies experienced clinically beneficially improvements in pain, disability and quality of life after minimally invasive SI joint fusion using a series of triangular porous plasma spray coated implants.

## Background

Pelvic girdle pain (PGP) is a term used to describe pregnancy-related pain in the sacroiliac joint (SIJ), lumbosacral region, pubic symphysis, or in any combination of these joints. PGP is widespread, affecting nearly half of all pregnant women (Vleeming et al. 2008; Wu et al. 2004). PGP can be distinguished from pregnancy related low back pain (PLBP) by its character, intensity, and location; PLBP is mainly described as a “dull ache” in the lumbar region while PGP is mostly described as pain between the posterior iliac crest and gluteal fold that may radiate into the thigh (Gutke et al. 2008; Vermani et al. 2010). While PGP will resolve in most women within 4 months after giving birth, approximately 20 % of women will experience persistent symptoms, regardless of culture or economic condition (Aldabe et al. 2012; Björklund and Bergström 2000; Ostgaard and Andersson 1992). Pelvic girdle pain that begins during pregnancy and does not resolve, or pain that develops soon after pregnancy is termed postpartum pelvic girdle pain (PPGP) (Vermani et al. 2010).

The exact underlying pathophysiology of PPGP remains unclear, although the most likely explanation is a combination of hormonal and biomechanical factors (Vermani et al. 2010). Hypermobility and ligamentous laxity of the SI joint due to increased levels of estrogen and relaxin appear in the third trimester of pregnancy (Dreyfuss et al. 2004). These altered hormonal factors allow the pelvic girdle to slightly expand in order to accommodate parturition. The most noticeable joint changes (identified radiographically) occur in the pubic symphysis. The joint widens and vertically shifts during pregnancy and delivery, with subsequent reduction after delivery (Björklund et al. 1999). Interestingly, neither increased relaxin levels peripartum nor degree of symphyseal distention have been shown to be a factors in the development of pelvic girdle pain (Aldabe et al. 2012; Björklund et al. 1999). Factors that have been shown to increase the risk of pelvic girdle pain include pre-pregnancy back pain (Sjödahl et al. 2013), back flexor weakness (Gutke et al. 2008), body mass index (Sjödahl et al. 2013), hypermobility, asymmetric SI joint ligament laxity (Damen et al. 2001), emotional distress (Bjelland et al. 2013), and vaginal delivery (Bjelland et al. 2013).

The current array of non-surgical PPGP treatments include medication optimization, physical therapy, and an individualized exercise regimen focused on pelvic stabilization (Vleeming et al. 2008). The *European Guidelines for the Diagnosis and Treatment of Pelvic Girdle Pain* do not recommend radiofrequency denervation; intra-articular SIJ injections are recommended only in the presence of ankylosing spondylitis (Vleeming et al. 2008). Neither of these have been specifically studied in the PPGP population.

Minimally invasive (MIS) SI joint fusion is an increasingly popular surgical treatment option for patients suffering from certain SI joint disorders (Rudolf 2012; Duhon et al. 2013; Sachs and Capobianco 2013; Graham Smith et al. 2013). Sacroiliac joint fusion investigation (SIFI, NCT01640353) is a prospective, multicenter single-arm clinical trial of MIS SI joint fusion using triangular-shaped titanium implants (iFuse Implant System^**®**^, SI-BONE, Inc., San Jose, CA, USA) in patients with SI joint disruption and/or degenerative sacroiliitis. The purpose of the present study is to assess the safety and effectiveness of MIS SI joint fusion in a subgroup of patients with degenerative sacroiliitis and/or SI joint disruptions whose pain began in the peri-partum period.

## Methods

### Patient population

At 26 centers across the US, 172 patients with diagnosed SI joint pain due to degenerative sacroiliitis and/or SI joint disruption were enrolled and treated in the SIFI study (NCT01640353) (Duhon et al. 2013). Participants were men and women between the ages of 21 and 70 who failed to achieve acceptable symptom relief after a minimum of 6 months of conservative care. Prior to study entry, patients were required to have undergone a complete diagnostic work up that included five physical examination maneuvers that stress the SI joint (Table 1) and an image-guided intra-articular SI joint block using local anesthetic. Eligibility required a positive response on 3 of 5 provocative maneuvers and at least 50 % reduction of SI joint pain after the injection. Further eligibility criteria included a score of at least 30 % on the Oswestry Disability Index (ODI) and a minimum SI joint pain rating of 50 mm on a 0–100 mm visual analog scale (VAS). The results of these examinations were recorded for the as baseline study parameters. Patients were excluded from study participation if they had severe low back pain as a result of diagnosed lumbar spinal pathology (e.g., lumbar disc degeneration, spinal stenosis, etc.), pain from known hip pathology, history of recent (<1 year) major trauma to the pelvis, or metabolic bone disease (either induced or idiopathic). Patients were also ineligible if they were receiving worker’s compensation or disability payments or involved in litigation for back or SI joint pain. After providing written informed consent, subjects underwent minimally invasive SI joint fusion using the iFuse Implant System as previously described (Rudolf 2012; Duhon et al. 2013). The study was IRB-approved at all clinical sites before enrollment began.

**Table 1:**
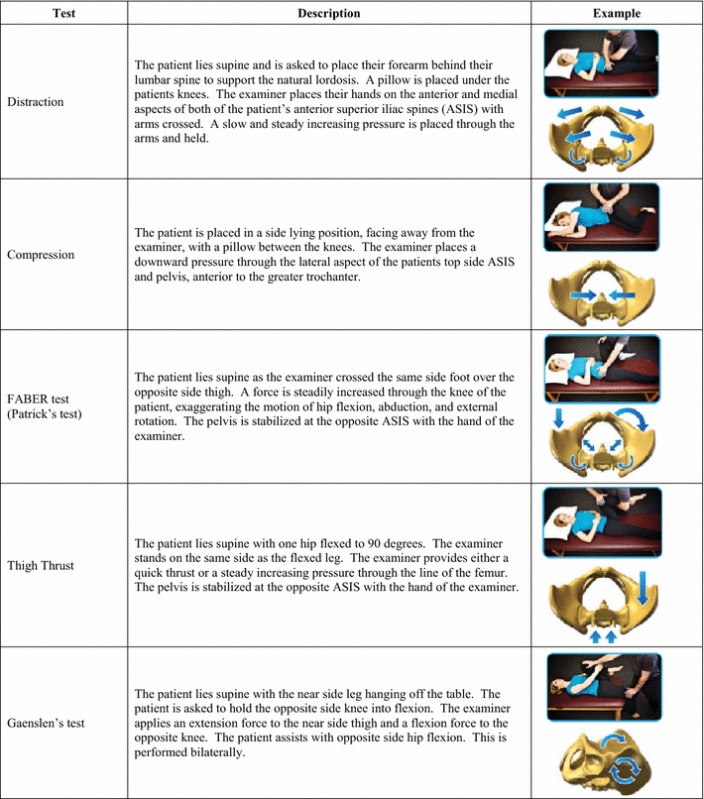
Physical provocative maneuvers

A medical history, neurological exam focused on the lower spine and pelvis, and a series of self-reported questionnaires were obtained at baseline. At postoperative follow-up visits, subjects underwent a neurological exam, adverse event assessment and were required to complete the same self-reported questionnaires. Self-reported questionnaires included SI joint and low back pain as measured on a visual analog scale (VAS), disability due to back pain using the Oswestry Disability Index (ODI), and quality of life using both EuroQol-5D (EQ-5D) and the Short Form-36 (SF-36). VAS was captured on a 0–100 mm unmarked scale with 0 representing no pain and 100 representing the worst pain imaginable. ODI is a validated ten-question survey for disability due to back pain. EQ-5D is a 5-question broad quality of life measure that can be combined into a single that represents the time trade-off (TTO) utility of current health (EuroQol Group 1990). SF-36 is a 36-question 8-subscaled generic quality of life measure that produces both a summary physical component score (PCS) and summary mental component score (MCS) (Ware and Sherbourne 1992). These measures are scaled such that the population norm is approximately 50 with a standard deviation of 10. Female subjects were classified as PPGP if they indicated that their pain began in the peripartum period. In the context of diagnosed SI joint pain, it is assumed that these women have pregnancy-related disruption or degeneration of the SI joint.

### Surgical procedure

**Fig. 1:**
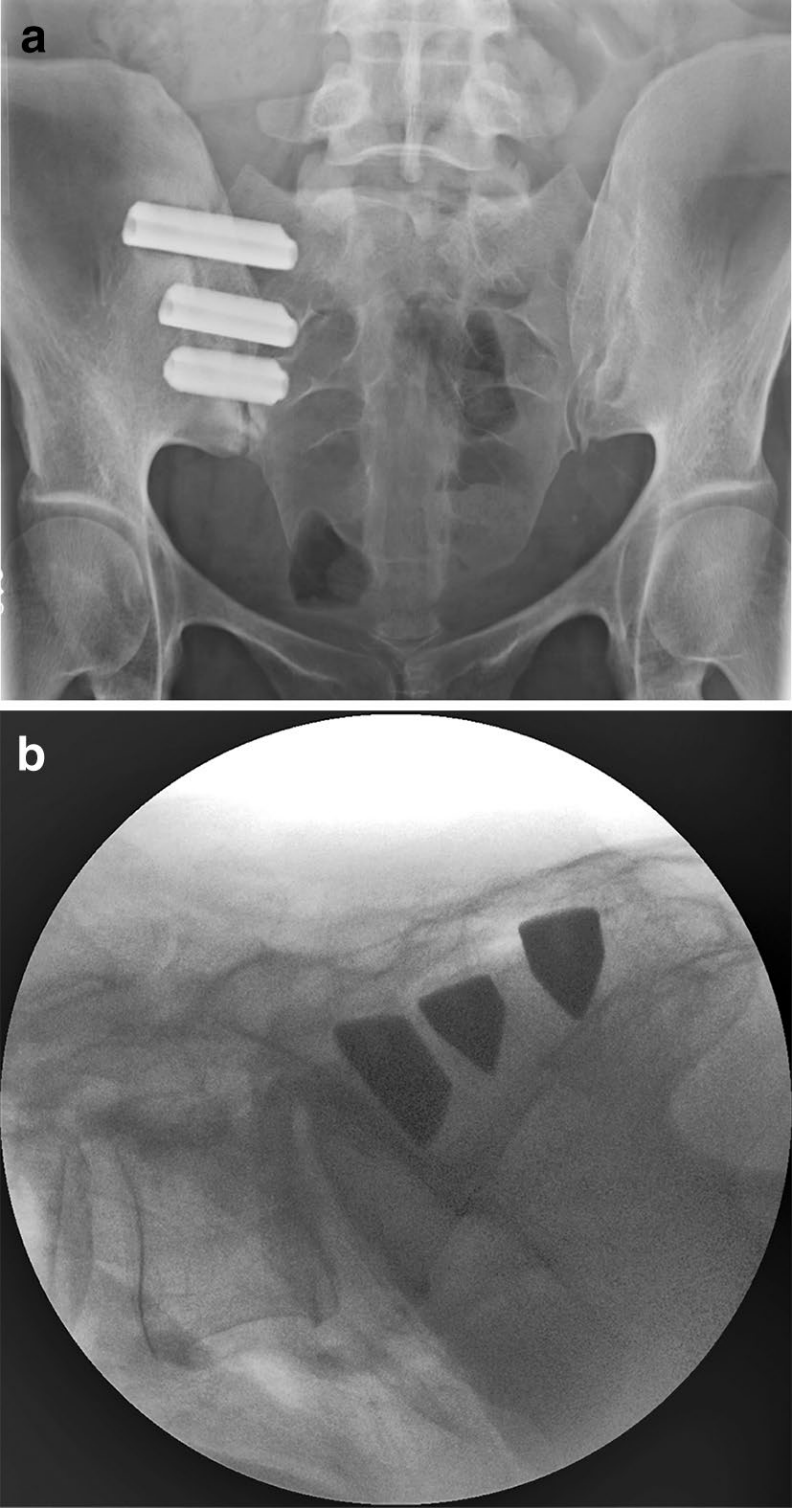
**a** Pelvic AP and **b** lateral plain film radiographs showing three implants placed across the SI joint

MIS SI joint fusion, performed in all subjects within 30 days of the baseline assessment, was completed under intraoperative fluoroscopic guidance. After general endotracheal anesthesia was administered, the patient was turned prone on a radiolucent table. A 3–5 cm lateral incision was made into the buttock region and the gluteal fascia was bluntly dissected to reach the outer table of the ilium. A guide pin was passed through the ilium across the SI joint to the center of the sacral ala, lateral to the neural foramen. A soft tissue protector was passed over the pin, and a drill was used to create a pathway into the sacrum and decorticate the bone. A triangular broach was then used to further decorticate the bone and prepare a pathway to receive the implant. Using a pin guidance system, additional implants were placed across the SI joint. In general, three implants are placed across the joint. The most cephalad implant was typically seated within the sacral ala above the S1 foramen. The second implant was generally located above or adjacent to the S1 foramen and the third between the S1 and S2 foramen (Fig. 1). The incision was irrigated and the tissue layers sequentially closed. Subjects who required treatment of both SI joints were offered either bilateral same-day or staged surgery. Perioperative measures, including estimated blood loss, fluoroscopy time, operating time, number of implants used, and complications, were collected.

Postoperatively, subjects were asked to remain at heel-toe touch-down weight-bearing using a front-wheeled walker or crutches for 3 weeks followed by progressive ambulation with crutches until fully ambulatory. Beginning 1–3 weeks postoperatively, subjects were asked to undergo individualized physical therapy twice a week for 6 weeks. Physical therapy involved activity modification to minimize pain recurrence, mobility and stability exercises, and adjacent segment joint mobilization for stiffness and pain control. Manipulation of the treated SI joint was discouraged.

### Outcome measures

The study’s primary endpoint was a binary success/failure composite endpoint at 6 months post-operatively. Success was defined if all of the following criteria were met: reduction in VAS SI joint pain by at least 20 mm, and absence of (1) serious device related adverse events, (2) neurological adverse events related to the study device, and (3) re-operation for any reason. Secondary outcome measures included change from baseline on VAS, ODI, EQ-5D, and SF-36 scores (individual components and PCS/MCS) at post-operative time points. Study follow-up will continue out to 2 years.

### Statistical analysis

An exact binomial confidence limit for the 6-month success proportion was calculated and compared with an estimate of the success rate with no treatment in this patient population, estimated at 35 %. A repeated measures analysis of variance was used to evaluate changes from baseline in pain, ODI, EQ-5D and SF-36. Logistic regression was used to compare the success rate in the PPGP group to both women without PPGP and men. Adverse event rates were tabulated. The number of adverse events per subject was compared with Poisson regression. All statistical analyses were performed using R (R Core Team 2013).

## Results

Of 172 subjects treated, 52 were male and 120 were female. Of the 120 women, 20 subjects (16.7 % of females, 11.6 % of all subjects) indicated that pain began in the peripartum period (Table 2). This constitutes the population designated as PPGP. Subjects with PPGP were significantly younger than both women without PPGP and men (mean 43.3 years vs. 52.5 and 50.7, respectively, p = 0.002). There was no difference in BMI (28.7) or duration of pain (6.3 years) between women with or without PPGP, or men. A slightly smaller proportion of PPGP patients (30 %) had previously undergone lumbar spinal fusion, compared to women without PPGP (42.2 %) and men (51.6 %, p = 0.206). Prior to study enrollment, all subjects had undergone a minimum 6-month course of conservative treatment and more than half of all patients underwent a course of physical therapy. More men than women with or without PPGP were treated with RF ablation prior to study entry (10 % PPGP, 14.4 % women without PPGP, 19.4 % men, p = 0.05). Baseline scores on all patient-reported outcome questionnaires did not differ between groups. Operative characteristics are summarized in Table 3.

**Table 2:** SIFI enrolled subject demographics

	PPGP (n = 20) mean (SD)	No PPGP (n = 100)	Men (n = 52)	
Age (years)	43.3 (9.0)	52.5 (11.1)	50.7 (11.4)	p = 0.002
BMI	28.7 (7.1)	29.4 (7.9)	29.5 (5.8)	p = 0.88*
Pain duration (years)	6.3 (7.4)	4.8 (5.3)	5.2 (7.2)	p = 0.62*
Prior lumbar fusion (%)	6 (30 %)	38 (42.2 %)	32 (51.6 %)	p = 0.21^†^
Prior treatments (n, %)				
Physical therapy	13 (65 %)	60 (66.7 %)	38 (61.3 %)	p = 0.79^†^
RF ablation	2 (10 %)	13 (14.4 %)	12 (19.4 %)	p = 0.05^†^
Steroid injections	20 (100 %)	87 (96.7 %)	55 (88.7 %)	p = 0.06^†^

* ANOVA

^†^Chi squared

**Table 3:** Operative characteristics

Characteristic	Value
Target joint, n (%)	
Right	83 (48.3 %)
Left	89 (51.7 %)
Procedure time, min	
Mean (SD, range)	46.4 (16.1)
Fluoroscopy time, min	
Mean (SD, range)	2.7 (1.8)
Estimated blood loss, cc	
Mean (SD, range)	51 (76)
Hospital length of stay, days	
Mean (SD, range)	0.8 (0.97, 0–7)
Discharged same day	69 (40.1 %)
1	85 (49.4 %)
2	10 (5.8 %)
3 or more	8 (4.7 %)

**Fig. 2:**
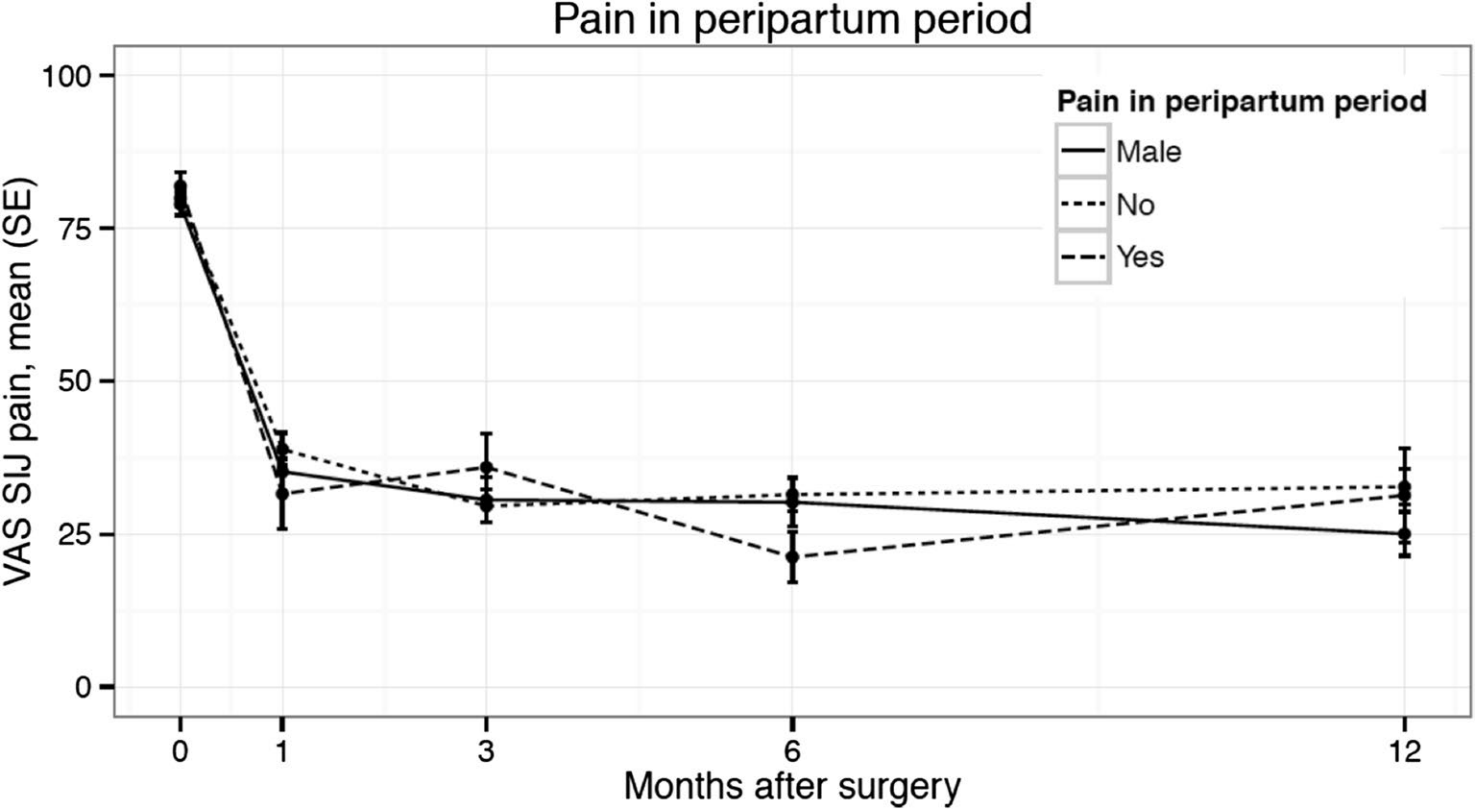
Mean VAS SI joint pain by time across subgroups

**Fig. 3:**
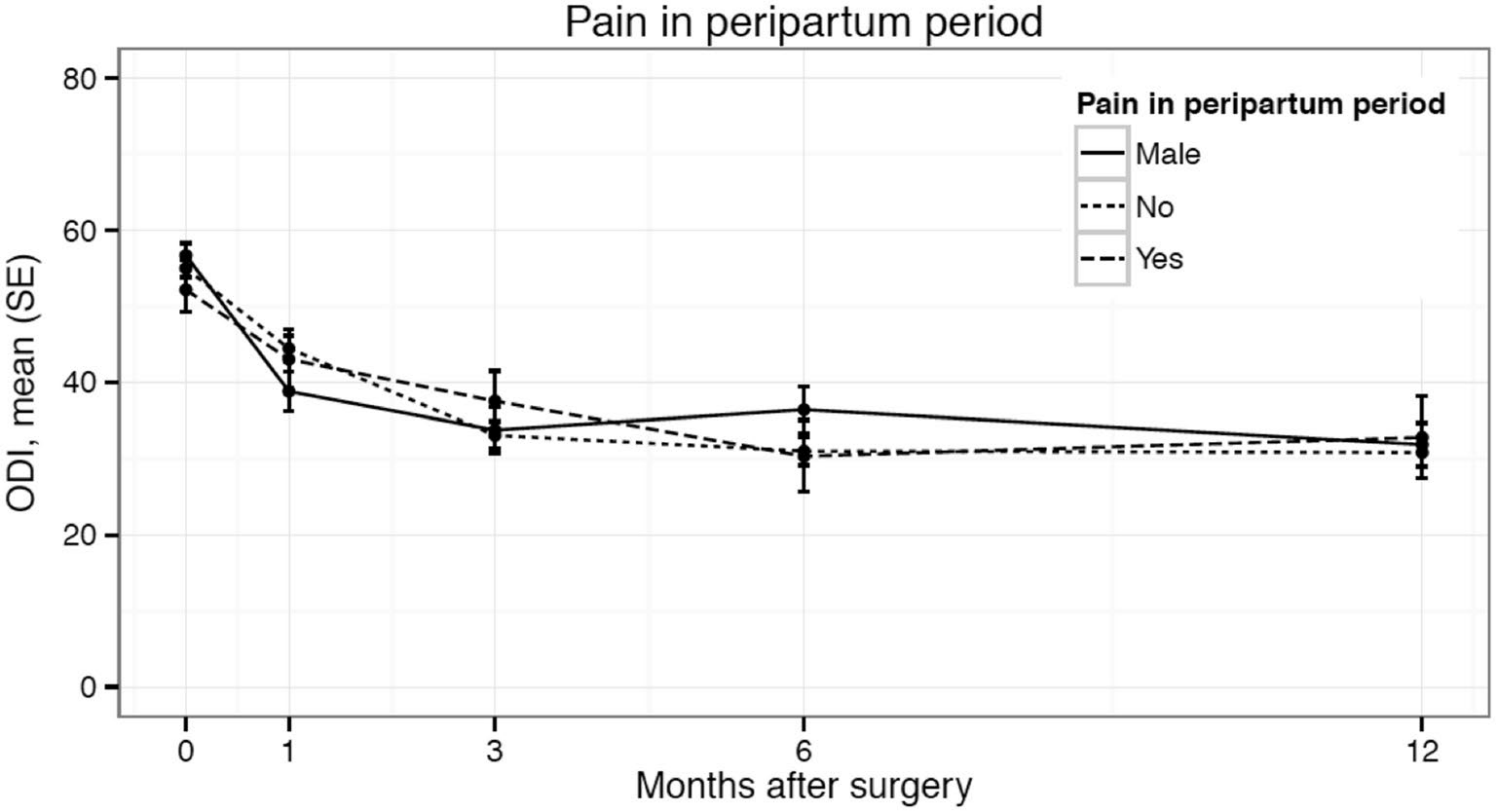
Mean Oswestry Disability Index by time across subgroups

**Fig. 4:**
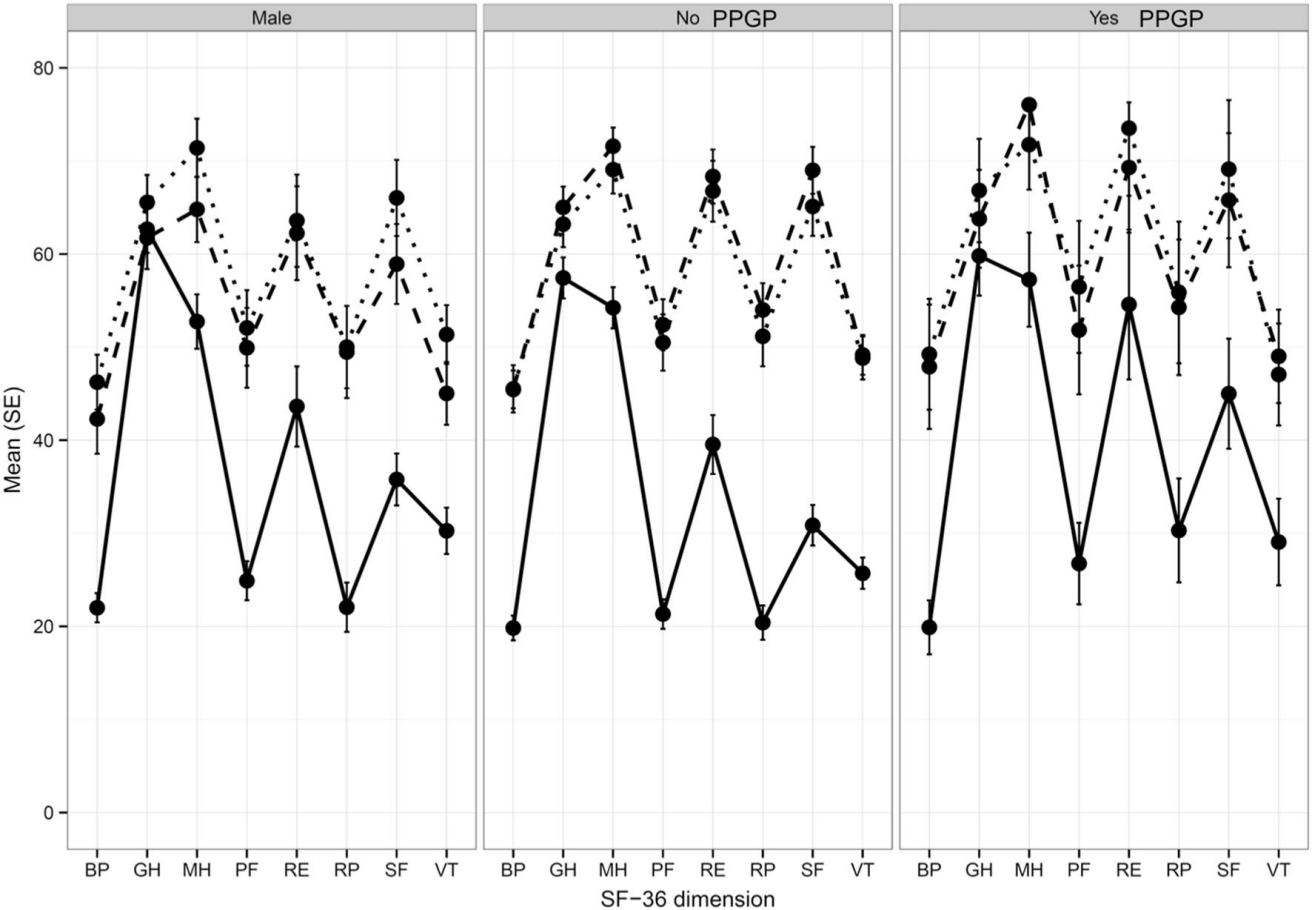
SF-36 scores by domain across subgroups

Women with PPGP experienced a significant improvement in pain, function and quality of life after SI joint surgery (Table 4). Compared to the baseline mean VAS pain level (81.9), pain in the PPGP subgroup was reduced by 51.1 points at 12 months (0–100 mm scale, p < .0001) (Fig. 2). Minimal clinically important difference, defined as a ≥20 mm change from baseline, was observed in 94.7 % of PPGP subjects. Disability measured on ODI improved by 20.6 points at 12-months; the mean score decreased from 52.2 at baseline to 30.4 at 6 months and 32.8 at 12 months (p < .0001) (Fig. 3). The physical component score (PCS) of the SF-36 increased significantly (higher scores represent better quality of life) from 32 at baseline to 40 at 6-months and 41.6 at 12-months (p = .002), reflecting an improvement in quality of life of approximately 1 SD (Fig. 4). Mean EQ-5D time trade-off (TTO) utility score improved from 0.42 at baseline to 0.72 at both 6- and 12-months, an improvement of 0.31 points (p < .0001). All of these improvements were typical of the larger study population. EQ-5D TTO, SF-36 PCS and MCS showed statistically significant effects across groups. Nearly all patients were very or somewhat satisfied with surgery at 1 year post-operatively (100 % PPGP, 84.0 % women without PPGP, 91.3 % men) and reported they might or definitely would have surgery again (94.1 % PPGP, 89.4 % women without PPGP, 93.5 % men).

**Table 4:** Patient reported outcomes

Study success	PPGP		No PPGP			Men
	N	Successful (%)	N	Successful (%)	N	Successful (%)
Success at 6 months	19	18 (94.7 %)	100	78 (78.0 %)	50	40 (80.0 %)
Success at 12 months	17	13 (76.5 %)	95	74 (77.9 %)	46	38 (82.6 %)

	PPGP		No PPGP		Men	
	N	Mean (SD)	N	Mean (SD)	N	Mean (SD)
VAS SI pain						
Baseline	20	81.9 (10.0)	100	79.9 (13.3)	52	78.9 (12.9)
1 month	20	31.6 (25.3)	98	38.9 (26.7)	50	35.2 (26.2)
3 months	20	36.0 (24.4)	99	29.6 (26.3)	50	30.6 (26.0)
6 months	19	21.3 (17.6)	100	31.5 (27.0)	50	30.2 (28.0)
12 months	17	31.4 (30.9)	94	32.7 (28.5)	45	25.0 (24.0)
12 month change		−51.1 (32.6)		−46.9 (29.9)		−52.9 (27.5)
>20 mm decrease	18	94.7 %	78	78 %	40	80 %
Change from baseline (p = .3708)^§^		p < .0001*		p < .0001		p < .0001
ODI						
Baseline	20	52.2 (12.7)	100	55.0 (11.2)	52	56.7 (11.5)
1 months	20	43.0 (16.9)	98	44.5 (16.9)	50	38.8 (18.3)
3 months	20	37.6 (17.3)	99	33.1 (17.8)	50	33.8 (21.2)
6 months	19	30.4 (20.0)	100	31.0 (18.7)	50	36.4 (21.4)
12 months	17	32.8 (21.4)	95	30.8 (19.1)	46	31.9 (18.9)
12 month change		−20.6 (26.0)		−24.1 (19.5)		−24.6 (21.0)
Change from baseline (p = .3100)		p < .0001		p < .0001		p < .0001
SF-36 PCS						
Baseline	20	32.0 (5.6)	98	31.1 (5.6)	51	32.7 (5.5)
6 months	19	40.0 (11.1)	100	40.5 (9.2)	49	39.8 (10.1)
12 months	17	41.6 (10.8)	91	40.0 (9.6)	46	40.5 (8.9)
12 month change		10.4 (10.1)		8.7 (9.9)		8.1 (9.8)
Change from baseline (p = .3623)		p < .0001		p < .0001		p < .0001
SF-36 MCS						
Baseline	20	42.2 (12.4)	98	37.7 (11.6)	51	38.6 (10.3)
6 months	19	49.7 (9.6)	100	48.8 (10.8)	49	45.1 (13.2)
12 months	17	49.0 (10.8)	91	47.7 (12.9)	46	48.0 (12.1)
12 month change		7.2 (12.0)		10.2 (11.9)		8.2 (11.2)
Change from baseline (p = .1313)		p < .0001		p < .0001		p < .0001
EQ-5D TTO						
Baseline	20	0.42 (0.14)	97	0.43 (0.18)	52	0.45 (0.19)
6 months	19	0.72 (0.23)	98	0.70 (0.19)	50	0.64 (0.25)
12 months	17	0.72 (0.21)	92	0.70 (0.20)	46	0.72 (0.19)
12 month change		0.31 (0.29)		0.27 (0.24)		0.26 (0.24)
Change from baseline (p = .0446)		p < .0001		p < .0001		p < .0001

	PPGP		No PPGP		Men	
	N					
Satisfaction at 12 months (p = .1252)						
Very or somewhat satisfied	17	100 %	79	84.0 %	42	91.3 %
No	0	0 %	15	16.0 %	4	8.7 %
Would have surgery again (p = .6503)						
Might or definitely yes	16	94.1 %	84	89.4 %	43	93.5%
No	1	5.9 %	10	10.6 %	3	6.5 %

* Using repeated measures analysis of variance

^§^Using repeated measures analysis of variance or chi-squared test

Adverse events, defined according to an international clinical trial standard (ISO14155: 2011), were collected at every study time point as well as any time the subject was in contact with the investigator’s office. Investigators were asked to determine the severity and relationship to the study device and surgical procedure for each event. Severity was classified as mild, moderate or severe. Relatedness was categorized as definitely, probably, possibly, unlikely or unrelated to the device or procedure. A total of 283 adverse events occurred between enrollment and the 12-month visit: 37 in women with PPGP, 158 in women without PPGP and 88 in men (Table 5). Most adverse events were related neither to the study device or study procedure. There was no difference in the mean number of events per subject between groups. Across the entire study population, 21 events were classified as *definitely or probably related* to the device or procedure: 4 in the PPGP group, 10 in women without PPGP and 7 in men. In the PPGP group, two subjects experienced a post-surgical wound infection. One subject complained of numbness at the surgical site. At 4 months after surgery, this same subject presented with pain secondary to a fall. CT imaging showed inadequate placement of the second and third implants. The subject was brought back to the operating room 13 months after the index procedure and one additional implant was placed. This subject subsequently experienced relief of symptoms. In the remaining subjects without PPGP, three subjects underwent subsequent revision (two women without PPGP, one man). Two revisions were immediate, in that subjects were brought back to the operating room soon after surgery in order to retract an implant that had violated the sacral neural foramen, resulting in symptomatic nerve impingement. One subject presented with pain at 4 months after the index surgery. CT scan revealed the third implant was not across the joint. The implant was replaced with a longer device and one additional implant was placed. The revision rate was 5 % in PPGP subjects, 2 % in women without PPGP and 1.9 % in men. The revision rate for all subjects in SIFI was 2.3 % (4/172). No subject experienced systemic complications, deep venous thrombosis or pulmonary embolism.

**Table 5:** Adverse events at 12-months

	PPGP	No PPGP	Male
Total number of events	37	158	88
Event rate per subject, mean (SD)	1.8 (1.3)	1.6 (1.4)	1.7 (1.6)
Related to device/procedure	4	10	7
Wound infection	2		2
Buttock pain	0	2	1
Postoperative neuropathy	0	1	1
Postoperative nausea/vomiting	0	3	0
SI joint pain	0	0	2
Intraoperative hemorrhage	0	1	0
Numbness around surgical wound	1	0	0
Neuropathy after contralateral SI joint fusion revision	0	1	0
Staple irritation	0	0	1
Urinary retention	0	1	0
Wound drainage	0	1	0
Fall causing SI joint pain. Poor device placement	1	0	0

## Discussion

Roughly 20 % of women who experience back pain during pregnancy report persistent pain 2 and 3 years postpartum (Norén et al. 2002). A substantial proportion of pregnancy- and post-partum-related back pain originates in and around the SI joint. The SI joint is a highly innervated diarthroidal joint subject to high mechanical stresses, especially during the peripartum period. Its exact innervation is quite variable both within and between individuals. In general, innervation of the SI joint is provided by various branches emanating anywhere from L5-S4 (Vleeming et al. 2012; Dreyfuss et al. 2003). These same segments also innervate muscles of the buttocks, hips and lower limbs. Furthermore, pain neurotransmitters, substance P, and calcitonin gene-related polypeptide have been detected in the cartilage of both sides of the joint as well as in the surrounding ligaments (Vleeming et al. 2012). Multisite injections of the sacral nerve roots can block some, but not all, elicited SI joint pain (Dreyfuss et al. 2009). Clearly, there is a neuropathway for pain perception in the region of the SI joint as well as low back, buttock and groin.

SI joint disorders associated with pregnancy are likely multifactorial and can be caused by a combination of hormonal, biomechanical, traumatic and degenerative factors. The design of the pelvic girdle provides the human body with both the stability needed to support the weight of the axial skeleton and the mobility required to accommodate habitual bipedal locomotion and childbirth. The sacrum has been described as “floating” in the pelvic girdle, stabilized by an intricate web of strong ligaments. The unique shape of the auricular surfaces, characterized by complimentary billowing and pitting, along with the basket weave of ligaments, creates self-bracing of the joint, referred to as form closure (Vleeming et al. 1992). Force closure, characterized by muscle forces and ligament tension, is maintained through a myofascial network spanning from the torso to the lower extremities. A small degree of mobility is observed via nutation and counternutation of the sacrum and vertical translation and compression of the pubic symphysis (Becker et al. 2010). Ligament loosening as a result of increased relaxin during pregnancy can create a cascade of events that can lead to degeneration or disruption of the SI joint. Ligaments are viscoelastic structures that creep and deform under constant load. Asymmetric ligament laxity will affect load transfer patterns, causing an imbalance in forces (Aldabe et al. 2012). Additionally, pain in the SI joint can lead to inefficient muscle recruitment, preventing necessary force closure of the joint to maintain stability (Agarwal et al. 2014).

Musculoskeletal structures are inherently unstable, allowing for agility critical to survival. The risk–benefit ratio is precariously held in balance—repeated assaults to the stability mechanism can have long-term degenerative effects (Gracovetsky 2007). A woman’s center of gravity shifts anteriorly both during pregnancy and post-partum while breast feeding, front baby carrying, and as a result of lifting, bending and lowering forces encountered during daily activities of caring for an infant. The constant shift in the center of gravity may result in an anterior rotation of the innominates, potentially leading to a loosening of the force couple, a decrease in intraarticular friction, and possible disruption or subsequent accelerated degeneration of the SI joint (DonTigny 2007).

Few effective non-surgical options are available for women with chronic, persistent PPGP related to the SI joint (Vleeming et al. 2008). The largest body of available literature describes outcomes after physical therapy (PT) interventions. Dysfunction of load transfer in the pelvis as well as delayed onset of muscle activation has been well described as a possible explanation of PGP (Mens et al. 2001; Hungerford et al. 2003; Snijders et al. 1993). This suggests that improvement in muscle activation and strength can alleviate pain. The current literature describes conflicting results. Four studies examined the effect of an intervention consisting of specific muscle stabilizing exercises. One randomized study found that while active care consisting of stabilizing exercises, a pelvic belt and education appears to be effective in alleviating pain during pregnancy (Elden 2005), these same interventions did not prevent post-partum PGP (Elden 2005; Elden et al. 2008). In another randomized trial comparing a program of various specific training exercises to no exercise, no difference was found between groups. Furthermore, while 64 % improved regardless of intervention, 36 % either had no change in symptoms or felt worse after the treatment (Mens et al. 2001). One study found a 20-week program of specific stabilizing exercises to be effective and durable at relieving pain out to 2-years postpartum (Stuge et al. 2004). However, a more recent study reported an individualized exercise program to be ineffective at relieving pain and back-related disability in women with persistent postpartum pelvic girdle pain, with or without concomitant low back pain (Gutke et al. 2010). Thus the literature on physical therapy for PGP and PPGP conveys not only an unclear message about specific stabilizing exercises, but unclear guidance as to which exercises produced favorable results. It is clear that some women may benefit from this approach, but many will have continued pain and degradation in quality of life.

Controlled randomized trials of SI joint injections, both periarticular (methylprednisolone vs. sodium chloride) (Luukkainen et al. 2002), and intra-articular (cartivazol vs. saline) (Maugars et al. 1996) show modest initial improvement in symptoms followed by a quick, marked decline towards baseline levels by 6 months. Similarly, both case series and sham-controlled trials of RF denervation report temporary improvement in less than half of subjects; success rates range from 36 to 47 % (Ferrante et al. 2001; Gevargez et al. 2002; Patel et al. 2012). None of these trials specifically enrolled women with PPGP as the cause of SI joint pain.

Surgical arthrodesis in the spinal column is a common and accepted treatment in the presence of pathology recalcitrant to conservative treatment modalities. Arthrodesis is the process of permanently joining 2 (or more) motion segments in order to eliminate pain caused by motion or abnormal loading of the joint. Success appears to largely depend on clear surgical indications and presence/absence of concomitant pathology and/or psychosocial factors (Spoor and Öner 2013). Pseudoarthrosis, or failure to achieve permanent stabilization, is a well-documented cause of persistent pain postoperatively (Raizman et al. 2009). In a similar vein, fusion of the SI joint has been attempted. The first published report of SI joint fusion through a lateral approach was reported in 1921 (Smith-Petersen 1921). Since that time, the body of literature on open SI joint fusion has grown modestly and documents varying degrees of clinical effectiveness. Compared to more recent reports of minimally invasive approaches, open surgical techniques are associated with relatively long operative times, lengthy hospital stays, considerable soft tissue dissection, and non-weight bearing for long periods of time (3 months). For these reasons, open surgery of the SI joint is usually reserved for the most severe and complicated cases.

Several implants using a variety of MIS techniques are currently available for MIS SI joint fusion. SIFI is a prospective multicenter clinical trial of one such device (iFuse Implant System). A reasonably sized subgroup of patients in this study (17 % of women) reported pain onset in the peripartum period. These women showed improvements in pain, disability and quality of life after MIS SIJ fusion that were similar to both women without PPGP and men. Improvements in pain were clinically significant; 94.7 % achieved pre-defined success in pain improvement and the overall mean improvement was three times larger than that considered to be clinically meaningful (20-point improvement) (Copay et al. 2008). Similarly, improvements in function and quality of life were substantial, exceeding commonly acceptable values. The rate of adverse events in women with PPGP was reasonably low.

The study reported herein has limitations. First, SIFI was not designed to diagnose PPGP. Some women who reported pain onset during pregnancy could have SI joint degeneration related to both peripartum disruption of the joint as well as subsequent joint degeneration as a result of age-related osteoarthritis or other factors such as adjacent segment degeneration after lumbar fusion (Weil et al. 2008). No longitudinal studies have investigated the long-term effects of peripartum SI joint disruption, but it is reasonable to assume that a large portion of affected patients will experience sequelae. Second, the number of subjects with PPGP was fairly low, limiting the ability to draw precise conclusions regarding differences in pain and QOL responses compared to the other subgroups. Nonetheless, no marked differences in pain relief and QOL improvement were seen across groups. Third, the study lacked a concomitant control group of women who received only non-surgical treatment. It is noted that all study participants had chronic (>6 months), carefully diagnosed SI joint pain and many had failed conservative treatment (medications, physical therapy, and, in some cases, RF ablation). Moreover, the mean duration of SI joint pain in PPGP subject was 6.3 years, suggesting both that treatments to date had not adequately relieved pain and that spontaneous pain relief with continued non-surgical treatment would have been unlikely. The findings reported herein are encouraging and represent the first report of minimally invasive surgical treatment for unremitting pain caused by SI joint dysfunction due to disruption or degeneration and associated with pregnancy. Physicians are encouraged to investigate the sacroiliac joint as a potential pain generator in patients presenting with persistent pelvic girdle pain. Moreover, further studies of minimally invasive SI joint fusion for the relief of post-partum-related SI joint pain are warranted.

## Conclusion

The sacroiliac joint is a source of pain in approximately 75 % of women with persistent PPGP. Physical therapy, the current mainstay of treatment, is an inconsistent therapeutic option that may provide relief for some women and has been shown to exacerbate symptoms in others. For carefully selected women with chronic post-partum related SI joint dysfunction recalcitrant to conservative therapies, MIS SI joint fusion may be a surgical option that has been shown to provide improvement in pain, disability and quality of life.

## References

[CR1] Agarwal Y, Doebele S, Windolf M (2014). Two-leg alternate loading model—a different approach to biomechanical investigations of fixation methods of the injured pelvic ring with focus on the pubic symphysis. J Biomech.

[CR2] Aldabe D, Milosavljevic S, Bussey MD (2012). Is pregnancy related pelvic girdle pain associated with altered kinematic, kinetic and motor control of the pelvis? A systematic review. Eur Spine J.

[CR3] Becker I, Woodley SJ, Stringer MD (2010). The adult human pubic symphysis: a systematic review: the pubic symphysis. J Anat.

[CR4] Bjelland EK, Stuge B, Engdahl B, Eberhard-Gran M (2013). The effect of emotional distress on persistent pelvic girdle pain after delivery: a longitudinal population study. BJOG Int J Obstet Gynaecol.

[CR5] Bjelland EK, Stuge B, Vangen S (2013). Mode of delivery and persistence of pelvic girdle syndrome 6 months postpartum. Am J Obstet Gynecol.

[CR6] Björklund K, Bergström S (2000). Is pelvic pain in pregnancy a welfare complaint?. Acta Obstet Gynecol Scand.

[CR7] Björklund K, Nordström ML, Bergström S (1999). Sonographic assessment of symphyseal joint distention during pregnancy and post partum with special reference to pelvic pain. Acta Obstet Gynecol Scand.

[CR8] Copay AG, Glassman SD, Subach BR (2008). Minimum clinically important difference in lumbar spine surgery patients: a choice of methods using the Oswestry Disability Index, medical outcomes study questionnaire short form 36, and pain scales. Spine J Off J North Am Spine Soc.

[CR9] Damen L, Buyruk HM, Güler-Uysal F (2001). Pelvic pain during pregnancy is associated with asymmetric laxity of the sacroiliac joints. Acta Obstet Gynecol Scand.

[CR10] DonTigny RL (2007) A detailed and critical biomechanical analysis of the sacroiliac joints and relevant kinesiology: the implications for lumbopelvic function and dysfunction. Mov Stab Low Back Pain Essent. Role Pelvis, Second. Elsevier, pp 265–278

[CR11] Dreyfuss P, Yin W, Willard F, Carreiro J (2003). Sensory stimulation-guided sacroiliac joint radiofrequency neurotomy: technique based on neuroanatomy of the dorsal sacral plexus. Spine.

[CR12] Dreyfuss P, Dreyer SJ, Cole A, Mayo K (2004). Sacroiliac joint pain. J Am Acad Orthop Surg.

[CR13] Dreyfuss P, Henning T, Malladi N (2009). The ability of multi-site, multi-depth sacral lateral branch blocks to anesthetize the sacroiliac joint complex. Pain Med.

[CR14] Duhon B, Cher D, Wine K (2013). Safety and 6-month effectiveness of minimally invasive sacroiliac joint fusion: a prospective study. Med Devices Evid Res.

[CR15] Elden H (2005). Effects of acupuncture and stabilising exercises as adjunct to standard treatment in pregnant women with pelvic girdle pain: randomised single blind controlled trial. BMJ.

[CR16] Elden H, Hagberg H, Olsen MF (2008). Regression of pelvic girdle pain after delivery: follow-up of a randomised single blind controlled trial with different treatment modalities. Acta Obstet Gynecol Scand.

[CR17] EuroQol Group (1990). EuroQol—a new facility for the measurement of health-related quality of life. Health Policy Amst Neth.

[CR18] Ferrante FM, King LF, Roche EA (2001). Radiofrequency sacroiliac joint denervation for sacroiliac syndrome. Reg Anesth Pain Med.

[CR19] Gevargez A, Groenemeyer D, Schirp S, Braun M (2002). CT-guided percutaneous radiofrequency denervation of the sacroiliac joint. Eur Radiol.

[CR20] Gracovetsky S (2007) Stability or controlled instability? Mov Stab Low Back Pain Essent. Role Pelvis, Second. Elsevier, pp 279–294

[CR21] Graham Smith A, Capobianco R, Cher D (2013). Open versus minimally invasive sacroiliac joint fusion: a multi-center comparison of perioperative measures and clinical outcomes. Ann Surg Innov Res.

[CR22] Gutke A, Ostgaard HC, Oberg B (2008). Predicting persistent pregnancy-related low back pain. Spine.

[CR23] Gutke A, Sjödahl J, Oberg B (2010). Specific muscle stabilizing as home exercises for persistent pelvic girdle pain after pregnancy: a randomized, controlled clinical trial. J Rehabil Med.

[CR24] Hungerford B, Gilleard W, Hodges P (2003). Evidence of altered lumbopelvic muscle recruitment in the presence of sacroiliac joint pain. Spine.

[CR25] Luukkainen RK, Wennerstrand PV, Kautiainen HH (2002). Efficacy of periarticular corticosteroid treatment of the sacroiliac joint in non-spondylarthropathic patients with chronic low back pain in the region of the sacroiliac joint. Clin Exp Rheumatol.

[CR26] Maugars Y, Mathis C, Berthelot JM (1996). Assessment of the efficacy of sacroiliac corticosteroid injections in spondylarthropathies: a double-blind study. Br J Rheumatol.

[CR27] Mens JM, Vleeming A, Snijders CJ (2001). Reliability and validity of the active straight leg raise test in posterior pelvic pain since pregnancy. Spine.

[CR28] Norén L, Ostgaard S, Johansson G, Ostgaard HC (2002). Lumbar back and posterior pelvic pain during pregnancy: a 3-year follow-up. Eur Spine J Off Publ Eur Spine Soc Eur Spinal Deform Soc Eur Sect Cerv Spine Res Soc.

[CR29] Ostgaard HC, Andersson GB (1992). Postpartum low-back pain. Spine.

[CR30] Patel N, Gross A, Brown L, Gekht G (2012). A randomized, placebo-controlled study to assess the efficacy of lateral branch neurotomy for chronic sacroiliac joint pain. Pain Med Malden Mass.

[CR31] R Core Team (2013). R: a language and environment for statistical computing.

[CR32] Raizman NM, O’Brien JR, Poehling-Monaghan KL, Yu WD (2009). Pseudarthrosis of the spine. J Am Acad Orthop Surg.

[CR33] Rudolf L (2012). Sacroiliac joint arthrodesis-MIS technique with titanium implants: report of the first 50 patients and outcomes. Open Orthop J.

[CR34] Sachs D, Capobianco R (2013). Minimally invasive sacroiliac joint fusion: one-year outcomes in 40 patients. Adv Orthop.

[CR35] Sjödahl J, Gutke A, Öberg B (2013). Predictors for long-term disability in women with persistent postpartum pelvic girdle pain. Eur Spine J.

[CR36] Smith-Petersen MN (1921). Arthrodesis of the sacroiliac joint. A new method of approach. J Bone Jt Surg.

[CR37] Snijders CJ, Vleeming A, Stoeckart R (1993). Transfer of lumbosacral load to iliac bones and legs Part 1: biomechanics of self-bracing of the sacroiliac joints and its significance for treatment and exercise. Clin Biomech Bristol Avon.

[CR38] Spoor AB, Öner FC (2013). Minimally invasive spine surgery in chronic low back pain patients. J Neurosurg Sci.

[CR39] Stuge B, Laerum E, Kirkesola G, Vøllestad N (2004). The efficacy of a treatment program focusing on specific stabilizing exercises for pelvic girdle pain after pregnancy: a randomized controlled trial. Spine.

[CR40] Vermani E, Mittal R, Weeks A (2010). Pelvic girdle pain and low back pain in pregnancy: a review. Pain Pract Off J World Inst Pain.

[CR41] Vleeming A, van Wingerden JP, Dijkstra PF (1992). Mobility in the sacroiliac joints in the elderly: a kinematic and radiologic study. Clin Biomech.

[CR42] Vleeming A, Albert HB, Östgaard HC (2008). European guidelines for the diagnosis and treatment of pelvic girdle pain. Eur Spine J.

[CR43] Vleeming A, Schuenke MD, Masi AT (2012). The sacroiliac joint: an overview of its anatomy, function and potential clinical implications. J Anat.

[CR44] Ware JE, Sherbourne CD (1992). The MOS 36-item short-form health survey (SF-36). I. Conceptual framework and item selection. Med Care.

[CR45] Weil YA, Hierholzer C, Sama D (2008). Management of persistent postpartum pelvic pain. Am J Orthop Belle Mead NJ.

[CR46] Wu WH, Meijer OG, Uegaki K (2004). Pregnancy-related pelvic girdle pain (PPP), I: terminology, clinical presentation, and prevalence. Eur Spine J Off Publ Eur Spine Soc Eur Spinal Deform Soc Eur Sect Cerv Spine Res Soc.

